# A Comparative Analysis of Conventional Tracheotomy Versus Bjork Flap Tracheotomy

**DOI:** 10.7759/cureus.36646

**Published:** 2023-03-24

**Authors:** Bhushan Chauhan, Amarjeet Kumar

**Affiliations:** 1 Department of ENT, Maharishi Markandeshwar Institute of Medical Sciences and Research, Mullana, Ambala, IND

**Keywords:** bjork flap, tracheotomy, tracheoesophageal fistula, tracheal stenosis, trachea, visual analogue score, decannulation, stoma, complications, conventional

## Abstract

Background and objective

Tracheotomy is a surgical technique performed in the anterior neck in various circumstances, such as prolonged endotracheal intubation, acute or persistent upper airway blockage, for bronchopulmonary toilet, or in certain otolaryngologic surgical procedures. In this study, we aimed to compare conventional and Bjork flap tracheotomy in terms of operative duration, as well as intraoperative, immediate postoperative, and delayed postoperative complications.

Materials and methods

A prospective study was conducted at a tertiary care hospital. The selected patients undergoing tracheotomies were randomly classified into two groups: conventional (n=30) and Bjork flap (n=30).

Results

Our findings indicated no statistically significant difference (p≥0.05) in terms of demographic profile (age and gender) between conventional (mean age: 52.3 ±12.79 years, male-to-female ratio: 25:5) and Bjork flap (mean age: 56.4 ±12.24 years, male-to-female ratio: 24:6) groups. A similar trend was observed in patients with respect to the duration of time required to establish access to the airway in both groups (7.8 ±1.73 and 7.7 ±1.87 minutes respectively, p≥0.05). However, a marked difference (p≤0.05) was observed in visual analog scale (VAS) scores between conventional and Bjork flap patients for ease of tube change (5.8 ±1.02-7.2 ±1.13 and 2.4 ±0.51-2.9 ±0.12) and stomal care (5.6 ±1.14-7.0 ±1.12 and 2.0 ±0.16-2.6 ±0.11) on the second and seventh day respectively. The Bjork flap-treated tracheotomy patients showed significantly favorable outcomes (p≤0.05) in intraoperative (immediate bleeding: 43%), postoperative (primary hemorrhage: 0%, subcutaneous emphysema: 6.7%), and delayed postoperative complications (stomal granulation: 10%, stomal stenosis: 3%, tracheostomy tube blockage: 10%, stoma infection: 10%, and secondary hemorrhage: 0%) as compared to their counterparts who underwent conventional tracheotomy: immediate bleeding: 70%; primary hemorrhage: 26.7%, subcutaneous emphysema: 30%; stomal granulation: 70%, stomal stenosis: 10%, tracheostomy tube blockage: 70%, stoma infection: 73%, and secondary hemorrhage: 3%. There was no significant difference with regard to tracheal stenosis and decannulation (p≥0.05) between the groups. Of the 25 decannulated patients, 50% (n=15) were in the conventional group and 33.3% (n=10) belonged to the Bjork flap group.

Conclusion

Based on our findings, Bjork flap tracheotomy is associated with fewer complications than conventional tracheotomy and may be preferred over conventional tracheotomy for elective tracheotomy procedures in adults.

## Introduction

Tracheotomy is one of the oldest life-saving procedures, and it has been practiced since time immemorial [1]. It is a surgical procedure to create an opening in the anterior tracheal wall to facilitate respiration, thereby bypassing the upper respiratory tract in terminally ill patients with upper airway obstruction. Nevertheless, it is also electively done in emergency cases to facilitate or wean the patient from the ventilator and for clearing bronchopulmonary secretions to prevent aspiration [2,3].

Various techniques have been used for making a tracheal incision. Literature has documented procedures ranging from making a surgical opening in the trachea via an incision on the tracheal wall (tracheotomy), excision of a portion of the anterior wall cartilage (tracheostomy) to percutaneous dilatation methods under endoscopic guidance [4]. Conventional tracheotomy and Bjork flap tracheotomy are the most commonly used procedures. Notably, both these procedures are safe and reliable and have associated advantages and limitations depending on the patient’s condition and the severity of the disease [2].

The conventional tracheotomy is generally performed on anesthetized endotracheal intubated patients. The patient lies in the supine position and a roll is placed under the shoulders to extend the neck. Local anesthesia is infiltrated into the midline between the cricoid cartilage and at a point 1 cm above the sternal notch. A vertical incision (2-3 cm) is given midway between the sternal notch and the cricoid cartilage. The vertical incision is generally preferred to ensure enough exposure, reduced risk of bleeding, and better aesthetics. The platysma is incised and the strap muscles are retracted laterally. With upward thyroid isthmus retraction, it invariably clears the tracheotomy site, resulting in excellent exposure of the trachea. Thereafter, the incision is made in the pretracheal fascia. A transverse incision is made between cartilages 2 and 3 or 3 and 4 after testing for tracheostomy tube cuff leakage. The cannula is inserted after elevating the upper and lower edges of the tracheostomy with tracheal hooks. It must be secured by the surgeon’s hand until sutures and tracheotomy tapes are applied [5].

During the tracheotomy procedure, the inferiorly based tracheal flap first demonstrated by Bjork in 1952 has endured and stood the test of time among the several anterior tracheal wall flaps that have been recommended. However, certain specific complications are observed with Bjork flap tracheotomy, and it remains a subject of controversy [6,7]. Major complications include bleeding, clogging, or dislodging of the tracheal tube, tracheoesophageal fistula, and tracheal stenosis though they are not common [5]. The Bjork flap tracheotomy gained prominence back in 1960 as an alternative technique to a single tracheal incision. An inverted U-shaped incision (4-5 cm) is made through the second, third, or fourth tracheal rings. The lumen is not inhibited as the resultant flap is sutured to the skin [7].

The Bjork flap lowers the shear pressures that are created during coughing as the tracheostomy tube slips against the tracheal mucosa. Furthermore, the trachea is pulled up to the skin and fastened even if the stoma has not fully developed, which supposedly makes tracheostomy tube changes safer and simpler [8,9]. The drawbacks or limitations associated with Bjork flap tracheotomy include increased microbial infection or bacterial colonization of the stoma. Moreover, the probability of complications increases with increased procedural duration [10].

The complications and outcomes of both conventional and Bjork flap tracheotomies have been illustrated in the literature. However, there is scarce data in terms of a comparison between the two methods with respect to procedural gaps, manifestations, and outcomes. In light of this, this study aims to compare conventional and Bjork flap tracheotomies on varied patient parameters, such as intraoperative, postoperative, and delayed postoperative complications.

## Materials and methods

Study population

A prospective study was conducted in the Department of Otorhinolaryngology at a tertiary care hospital. Ethical clearance was obtained from the Institutional Ethical Committee of the hospital (IEC 1974). The study was conducted for a period of two years from September 2020 to September 2022.

Inclusion and exclusion criteria

The study included patients requiring tracheotomy for establishing the airway. The inclusion criteria were as follows: patients older than 18 years and younger than 75 years of age undergoing tracheotomy under general or local anesthesia in the department of ENT by the same surgeon. The exclusion criteria were as follows: patients undergoing tracheotomy with neoplasm on the tracheal wall, intra-luminal growth, deviated trachea and prior tracheotomized state were not considered for the study. Pregnant and lactating women or patients undergoing emergency tracheotomy were also excluded.

Sample size

The sample size was calculated using power analysis software with a margin of error of 5%, power of 0.8, and effect size d at 0.8. The patients fulfilling the inclusion criteria were selected (n=60) and classified via block randomization with a block of five into two groups, namely the conventional group (n=30) who underwent a conventional tracheotomy and the Bjork flap group (n=30) who underwent Bjork flap tracheotomy. Single blinding was conducted wherein the patients consented to both procedures; however, they were not aware as to which group they would be assigned.

Study design

Written informed consent was obtained from the selected patients. They were counseled and given a detailed explanation regarding the study, its objectives, procedure, and expected outcomes prior to the commencement of the study.

The patients were assessed for varied parameters at different time intervals, such as ease of procedure, tube change, stomal care, and tube insertion, as well as intraoperative (bleeding, tracheoesophageal fistula, and injury to the recurrent laryngeal nerve), immediate postoperative (primary hemorrhage, subcutaneous emphysema, accidental decannulation, pneumothorax), and delayed postoperative complications (stomal granulation, stomal stenosis, tracheoesophageal fistula, tracheocutaneous fistula, tracheostomy tube blockage, stoma infection, and secondary hemorrhage). The patients were followed up every week for routine general checkups for a period of one year. They were followed up at four months for tracheal stenosis. Decannulated patients were followed up for six months for tracheocutaneous fistula. The patients, their family members, and the nursing staff were explained and instructed about the visual analog scale (VAS) score. They were asked to score the responses for varied intraoperative and postoperative (second and seventh day) parameters on a scale ranging from 0 (very easy) to 10 (very difficult). A higher VAS score indicated greater difficulty for the patients. The bleeding was qualitatively assessed and the patients were marked as positive if there was significant blood loss during the procedure.

Statistical analysis

Statistical analysis was performed using Student’s t-test and Mann-Whitney U test to compare the differences between conventional and Bjork flap tracheotomy groups. The Chi-square test was used to evaluate the qualitative variables. A p-value ≤0.05 was considered statistically significant. All analyses were performed using IBM SPSS Statistics v 20.0 (IBM Corp., Armonk, NY).

Tracheotomy

The patients were subjected to open tracheotomies, namely conventional and Bjork flap tracheotomies.

Conventional Tracheotomy

**Figure 1:**
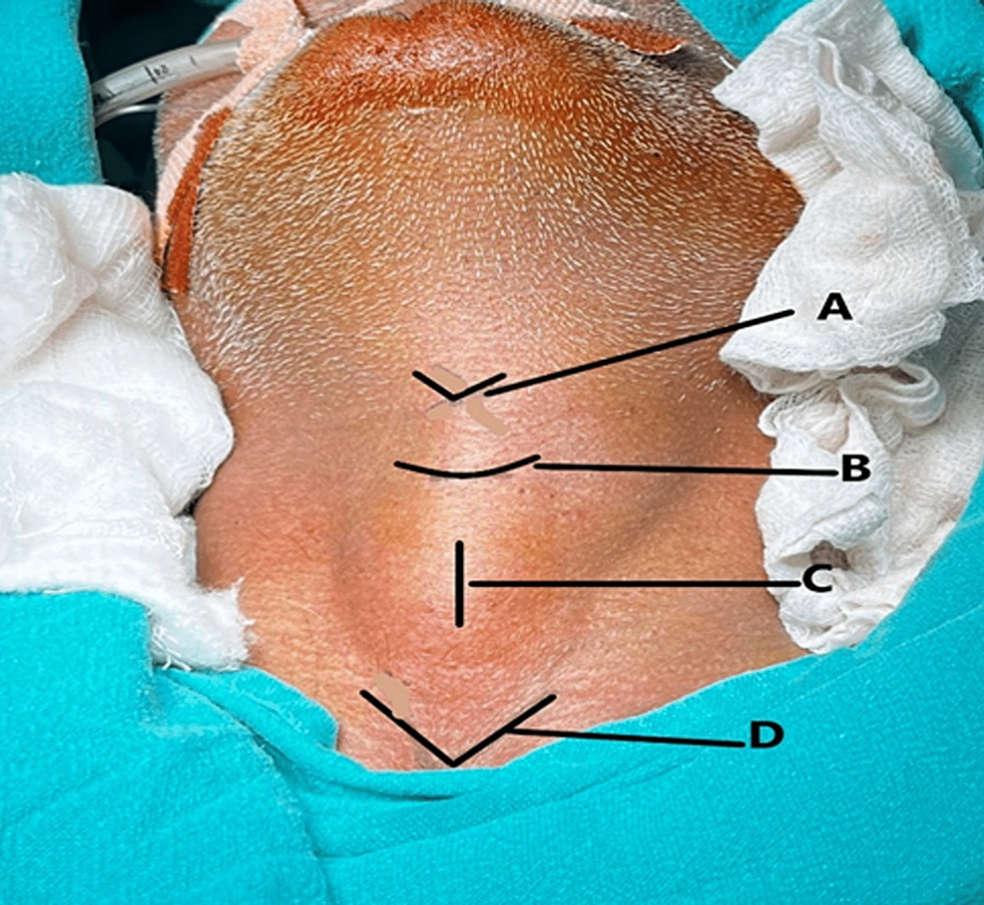
Landmarks for tracheotomy A: thyroid notch; B: cricoid cartilage; C: midline vertical incision marking; D: sternal notch

The patient was subjected to elective tracheotomy under a supine position and general anesthesia. Surgical landmarks from superior to inferior, namely the thyroid notch, cricoid cartilage, and sternal notch, were made. It involves a 2-3 cm vertical incision midway between the sternal notch and the cricoid cartilage (Figure 1).

**Figure 2:**
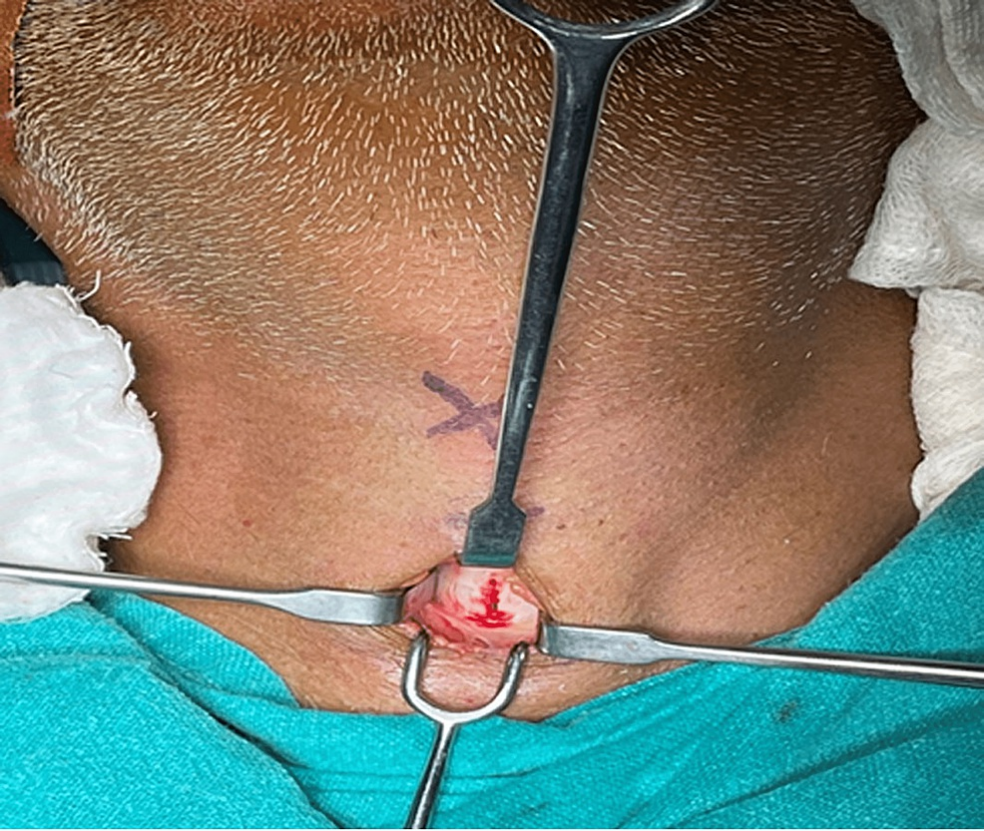
Tracheal incision between second and third tracheal ring

Following a vasoconstrictive local infiltration with 1% lidocaine and 1:100,000 epinephrine, electrocautery was used after incising subcutaneous fat and platysma. A midline raphe between the strap muscles was identified to proceed vertically while dissecting. The retractors were employed bilaterally to retract the strap musculature laterally. In order to identify and avoid injury to a high-riding innominate artery, a finger was put in the right inferior aspect of the wound bed. The midline dissection was continued until the thyroid gland was encountered. The thyroid isthmus was clamped, divided, and suture ligated. Furthermore, the division of the isthmus may not be necessary in every case if exposure of the trachea is enough by superior retraction of the thyroid gland. The cricoid cartilage was then palpated superiorly and pulled in an anterior-superior direction with the aid of a single, sharp hook after removing the loose pre-tracheal fascia to improve the exposure of the trachea (Figure 2).

**Figure 3:**
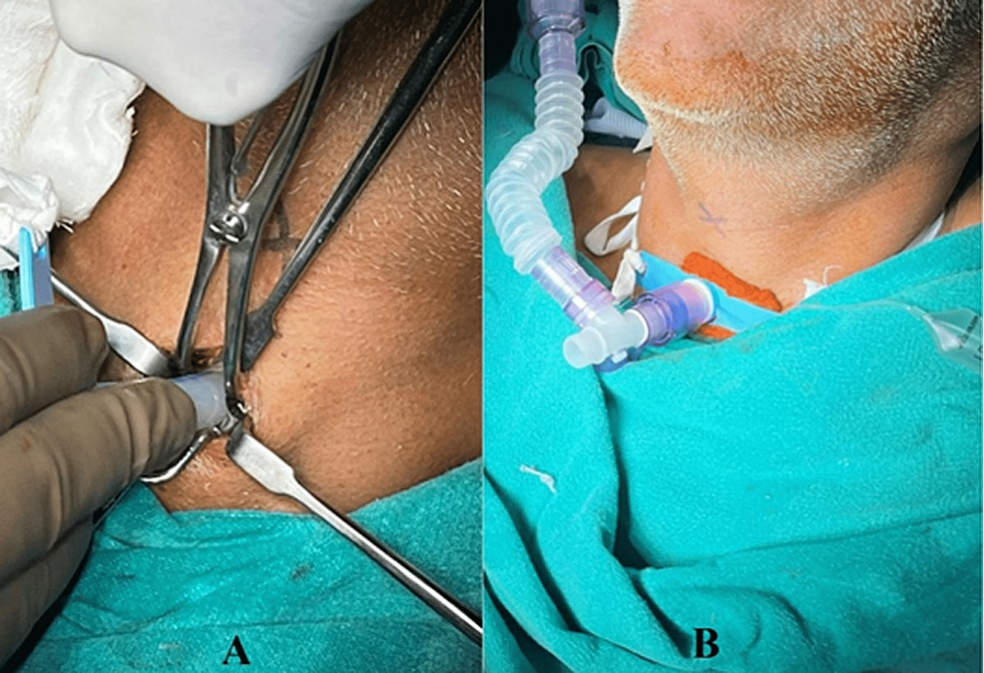
A: T-tube insertion; B: tube connected to the anesthesia circuit and secured with a soft trans-cervical tie

The tape or ties from the oral/nasal endotracheal tube were removed and secured manually. The inspired fraction of oxygen was reduced. The endotracheal tube's cuff was deflated to reduce the possibility of a balloon rupture during tracheotomy. Additionally, it was intended to tracheotomize between the second and third tracheal rings. To confirm the location of each tracheal ring, the cricoid cartilage was palpated with the finger. The oral/nasal tracheal tube was pulled back superiorly until the tip of the tube was observed just above the level of the tracheostomy. A vertical tracheal incision was made in the anterior tracheal wall between the second and third tracheal rings and an oval-shaped hole was made in the trachea with a No. 11 blade (Figures 3A, 3B).

The retractors and the endotracheal tube were left in place until the patient was adequately ventilated via the tracheostomy tube. This was ascertained with bilateral chest auscultation and the return of carbon dioxide from the respiratory circuit. Thereafter, both retractors and endotracheal tube were removed and the tracheostomy tube was secured by means of a tracheostomy tie around the neck and suture of the anterior neck at the four corners of the tracheostomy tube flanges [5].

Bjork Flap Tracheotomy

**Figure 4:**
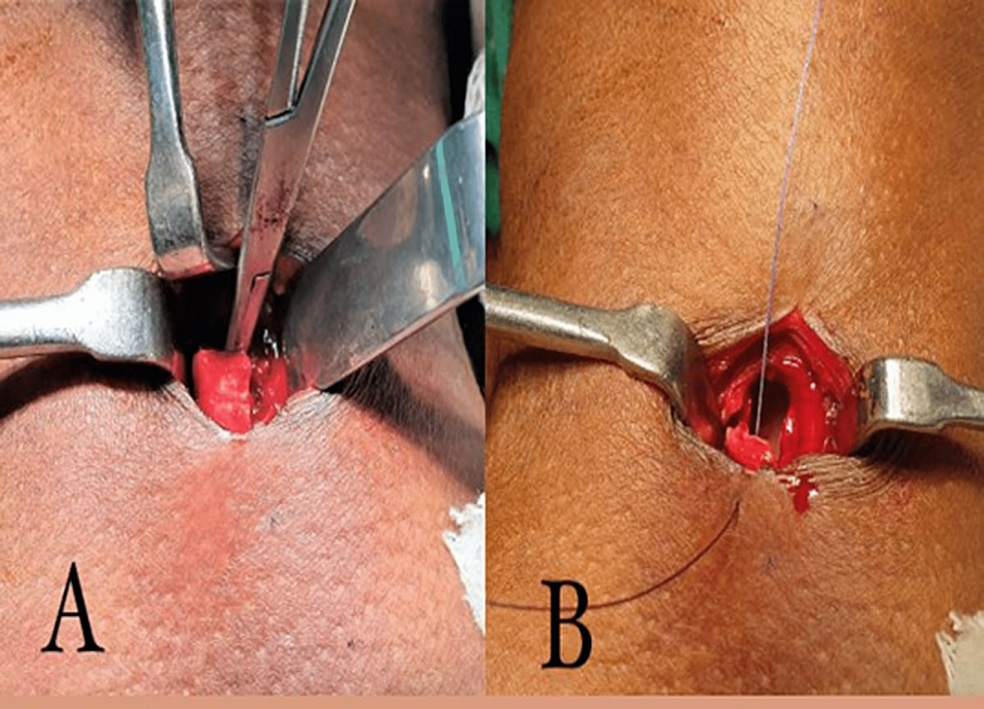
A: inverted U-shaped Bjork flap; B: Bjork flap sutured to the inferior margin of skin incision

Bjork Flap tracheotomy was done via a short (1.5 cm) vertical midline incision centered halfway between the suprasternal notch and the cricoid cartilage. The incision was made just long enough to fit the tracheostomy tube snugly and securely, thereby obviating the need for sutures and eventually for surgical closure of the tracheostomy. Kilner retractors with specifically adapted handles were used to provide sufficient retraction to enable and facilitate dissection through the small incision. A tracheostomy hook was used to lift up the trachea into the wound. An inverted U-shaped incision was made into the trachea through rings 2, 3, and 4. The inferiorly hinged flap of the anterior tracheal wall was secured to the inferior margin of the skin incision with sutures (Figures 4A, 4B) [10].

The tracheostomy tube was inserted into the stoma after withdrawing the endotracheal tube. Tracheostomy flanges were secured to the skin with 3-0 silk sutures. To prevent obstruction of venous return from the head and neck region, no circumferential tapes were applied to the neck. The tracheal cuff pressure was monitored using a pressure gauge (between 25-30 cm of water) after inserting the tube to ensure that the cuff's expansion did not raise the pressure because of increasing gas temperatures and nitrous oxide diffusion [10].

## Results

The study was planned to compare conventional tracheotomy with Bjork flap tracheotomy. A total of 60 patients undergoing tracheotomies, fulfilling inclusion criteria, and willing to participate in the study were selected. They were then equally divided by random block distribution with a block of five into two groups, namely the conventional group comprising 30 patients (50%) who underwent a conventional tracheotomy and the Bjork flap group comprising 30 patients (50%) who underwent Bjork flap tracheotomy.

The mean age of the patients in the Bjork flap group was higher (56.4 ±12.24 years) compared to the conventional group (52.3 ±12.79). However, this was not statistically significant (p≥0.05), as shown in Table 1.

**Table 1:** Comparison of mean age between conventional and Bjork flap tracheotomy patients SD: standard deviation

Treatment groups	Number of patients	Age in years, mean ±SD	P-value
Conventional	30	52.3 ±12.79	0.214
Bjork flap	30	56.4 ±12.24	

As shown in Table 2, the majority of the patients (n=32, 53.3%) were in the age group of 56-75 years. Furthermore, more patients in this age group (n=18, 56.3%) underwent Bjork tracheotomy than conventional tracheotomy (n=14, 43.7%). Gender-wise distribution indicated a male predominance (n=49, 81.7% vs. n=11 females, 18.3%) with males accounting for the majority of patients (n=17, 53.1%) in the Bjork flap group in the age category of 56-75 years. The statistical analysis revealed no significant difference (p≥0.05) in terms of age group and gender distribution.

**Table 2:** Comparison of gender-wise distribution between conventional and Bjork flap tracheotomy patients

Age groups (years)	Treatment groups				Total
	Conventional		Bjork flap		
	Male	Female	Male	Female	
18-36	-	3	1	1	5
37-55	12	1	6	4	23
56-75	13	1	17	1	32
Total	25	5	24	6	60

**Figure 5:**
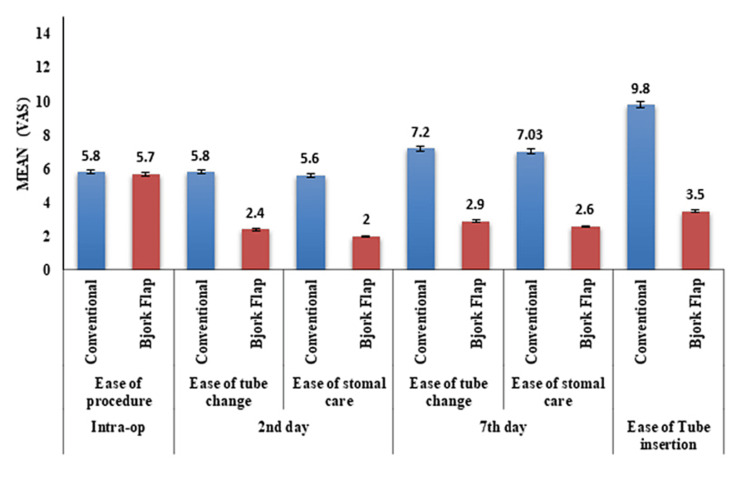
Comparison of mean parameters at varied intervals between conventional and Bjork flap tracheotomy patients VAS: visual analog scale

Figure 5 depicts the comparison of varied parameters, namely ease of procedure, tube change, stomal care, and tube insertion with respect to time intervals (second and seventh day). The assessment was made by the surgeon based on the inputs received from patients, their family members, and the nursing staff using VAS; the results showed that mean VAS scores were significantly higher (p≤0.05) in the conventional group at all procedural steps for varied parameters (ease of tube change, stomal care, and tube insertion) on the second and seventh day in comparison to Bjork flap group, indicating that Bjork flap tracheotomy was significantly better than a conventional tracheotomy.

The time required to establish airways was assessed in both groups. Results revealed no significant difference (p≥0.05) between the groups (7.8 ±1.73 and 7.7 ±1.87 minutes) indicating both the tracheotomy procedures were equally competent in terms of the time required for establishing airways. The difficulties encountered during the intraoperative stage such as thyroid isthmus present in the surgical field, short neck, morbid obesity, difficulty in identifying tracheal rings, and high riding innominate artery in patients undergoing both tracheotomies were observed in equal measure in both the groups (16.7%); however, the majority of patients (83.3%) in both groups reported no difficulty.

**Figure 6:**
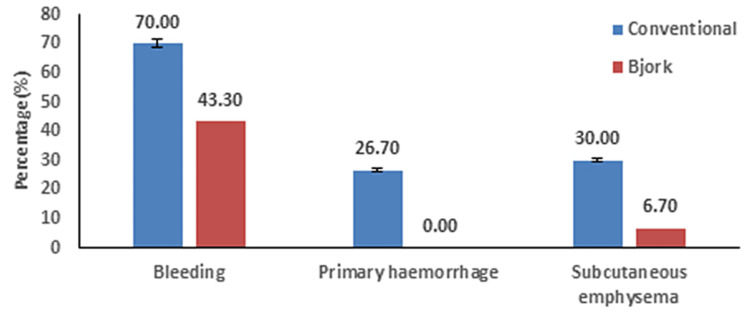
Comparison of intraoperative and postoperative complications between conventional and Bjork flap tracheotomy patients

Intraoperative complications were evaluated in terms of bleeding, tracheoesophageal fistula, and injury to the recurrent laryngeal nerve. The patient was marked positive for intraoperative bleeding if blood loss was significant and caused difficulty while performing the tracheotomy. Bleeding was significantly higher (p≤0.05) in the conventional group (n=21, 70%) in comparison to the Bjork flap group (n=13, 43.3%) (Figure 6). The other two complications were not reported in any of the patients in either group. Likewise, postoperative complications, namely primary hemorrhage, subcutaneous emphysema, accidental decannulation, and pneumothorax were assessed in patients of both groups; 26.7% of the patients in the conventional group had a primary hemorrhage, and 30% had subcutaneous emphysema (p≤0.05) (Figure 6). Conversely, only 6.7% of patients in the Bjork flap group showed subcutaneous emphysema (p≤0.05%). None of the patients in either group showed the other two postoperative complications (accidental decannulation and pneumothorax).

**Figure 7:**
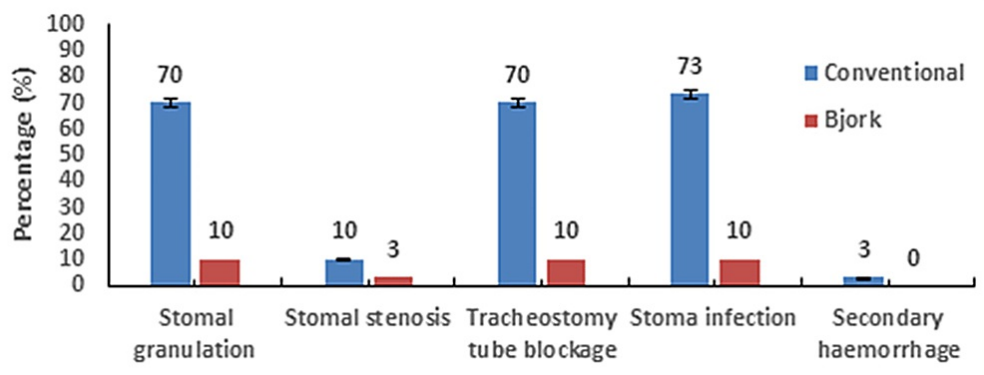
Comparison of delayed postoperative complications between conventional and Bjork flap tracheotomy patients

Additionally, delayed postoperative complications, namely stomal granulation, stomal stenosis, tracheoesophageal fistula, tracheocutaneous fistula, tracheostomy tube blockage, stoma infection, and secondary hemorrhage were assessed in both groups. Results showed a higher prevalence of delayed postoperative complications in the conventional tracheotomy group compared to the Bjork flap group. As shown in Figure 7, there was significant variation (p≤0.05) between groups with a higher prevalence of stomal granulation (70%), tracheostomy tube blockage (70%), and stoma infection (73.3%) in the conventional group. Secondary hemorrhage was only reported in the conventional group but the prevalence was low (3.3%). Notably, none of the patients showed any symptoms of a tracheoesophageal fistula or tracheocutaneous fistula in either group.

Patients were followed up at four months for tracheal stenosis, and a significant majority of patients (96.7%) reported no symptoms of tracheal stenosis in either group (p≥0.05). Decannulation was performed on 25 of the total 60 patients. Of the 25 decannulated patients, 50% (n=15) were in the conventional group and 33.3% (n=10) belonged to the Bjork flap group (p≥0.05) (Table 3).

**Table 3:** Comparison of tracheal stenosis and decannulation between conventional and Bjork flap tracheotomy patients

	Treatment groups		
Tracheal stenosis	Conventional	Yes	3.3%
		No	96.7%
	Bjork flap	Yes	3.3%
		No	96.7%
Decannulation	Conventional	Yes	50%
		No	50%
	Bjork flap	Yes	33.3%
		No	66.7%

## Discussion

Tracheotomy is a common operating procedure in otolaryngology (ENT) departments and ICUs. This prospective study was carried out to compare conventional and Bjork flap tracheotomy in terms of varied patient parameters such as operative duration and intraoperative, immediate postoperative, and delayed postoperative complications. Contrary to a standard tracheotomy, which removes the anterior tracheal wall window, Bjork flap tracheotomy involves a flap of the anterior tracheal wall that is secured to the inferior edge of the soft tissue incision by a non-absorbable suture. This prevents the flap from moving, falling back, and occluding the stoma. The resulting tract matures quickly and the endotracheal mucosa-lined inferior surface ensures appropriate curvature and smooth passage, making it simple and easier to insert the tube.

Prior research has shown that patients who had undergone Bjork flap tracheotomies did not exhibit any unattractive scar, postoperative tracheocutaneous fistula, stenosis, or mortality [10-11]. Similarly, another study that evaluated tracheal stenosis using endoscopy in Bjork flap patients reported that only 11% of patients exhibited narrowing of the tracheal lumen, and none of them had narrowing exceeding more than 50% [12]. Furthermore, Bjork flap tracheotomy patients (10%) showed a decreased rate of tracheal narrowing at the stoma with narrowing occurring at eight weeks after extubation in comparison to 95% of window patients [13].

The present study involved 60 patients undergoing a tracheotomy. The selected patients were randomly allocated to two groups, namely conventional and Bjork flap, with 30 patients in each group. A midline precise vertical skin incision was made in patients in both groups for gaining better access to deep structures, visualization, and protection of lateral structures to the trachea, such as the recurrent laryngeal nerves. Additionally, a sufficient but modest vertical incision eliminates the cosmetically objectionable scar [10]. Youseff et al. [14] have documented that surgical tracheotomy can lead to several complications, with an overall incidence of 6-66%, including pneumothorax or subcutaneous emphysema (4-17%), tube dislodgement (0-7%), bleeding (3-37%), stomal infection (17-36%), and a mortality rate of 0-5.3%.

The intergroup variation regarding the demographic profile (mean age and gender) of patients between the groups showed no statistical difference (p≤0.05). Similar results have been reported in another study that compared conventionally tracheotomized patients with those in whom a flap-based method was followed [15].

The VAS scores were used to assess the ease of procedure, tube change, stomal care, and tube insertion in patients of both groups. The results revealed similar mean VAS scores for both tracheotomies for the ease of procedure. The mean VAS scores of patients operated through conventional tracheotomy were significantly higher (p≤0.05) for other varied parameters at all procedural steps on the second and seventh day in comparison to the Bjork flap group, suggesting that Bjork flap tracheotomy has an edge over the conventional tracheotomy. Similar findings have been reported earlier [15]. The study illustrated that tube change was difficult in conventional tracheotomy patients and required the intervention and support of an ENT surgeon to the nursing staff. Conversely, Bjork flap-treated patients did not require any intervention from an ENT surgeon. Furthermore, the authors reported that the ease of stomal care was four times more favorable (76.36%) in patients operated via the Bjork flap method compared to the conventional method (16.36%) based on VAS scores. Weissler (2006) has demonstrated similar findings regarding Bjork flap tracheotomy [16]. Furthermore, Heffner et al. demonstrated that even in cases of unintentional or accidental decannulation, Bjork flap tracheotomy patients reported better ease of tube placement than their conventional tracheotomy counterparts [17]. Hence, it is safer to employ Bjork flap tracheotomy as it helps in providing a tracheal conduit bridge that directs the tracheostomy tube and prevents erroneous passage. Also, early maturity of the stoma is ensured by the flap-based treatment with improved mucosalization of the tract [15].

Surgical interventions are routinely associated with complications. Generally, a huge burden of complications is observed in tracheotomies, which needs to be minimized for a good postoperative recovery. The airway management in both tracheotomies showed similar duration. As for intraoperative complications, bleeding was more pronounced in patients who underwent a conventional tracheotomy. Nevertheless, the other two parameters - tracheoesophageal fistula and injury to recurrent laryngeal nerve - were not reported by any of the patients in either group. Our findings are consistent with the results of previous studies wherein the correct indication and timing of surgery and proper surgical technique and postoperative care have been reported to be the prerequisites for the prevention of tracheoesophageal fistula [18]. Additionally, another study that compared percutaneous and conventional tracheotomies reported that minimal to moderate bleeding was observed in both procedures; however, the former technique was more effective and safe when compared to the conventional method with a lower incidence of postoperative complications [14]. A similar trend was observed in terms of postoperative complications like primary hemorrhage and subcutaneous emphysema in conventional tracheotomy patients. This can be ascribed to surgical dissection, neck anatomy changes, coagulopathies, the severity of the disease, and the patient's general health status. Likewise, 9.09% and 7.27% of conventional tracheotomy-treated patients and 7.27% and 1.81% of Bjork flap-treated patients showed bleeding and surgical emphysema respectively [15]. Hence, our results are in line with the earlier findings indicating that Bjork flap tracheotomy is associated with fewer postoperative complications. Notably, the other postoperative complications - accidental decannulation and pneumothorax - were not observed in any of the patients in either group. Likewise, Youssef et al. have reported that among the total patients (n=64) in both groups in their study - percutaneous dilatational tracheostomy (PDT, n=32) and conventional surgical tracheostomy (CST, n=32) - no significant difference (p≥0.05) was observed with regard to patients developing pneumothorax. Furthermore, none of the patients in the PDT group displayed postoperative emphysema. Nevertheless, one patient in the CST group required close surveillance, which was resolved completely after three days. Significantly, none of the patients in either group reported any incidence of inadvertent decannulation or postoperative hemorrhage [14].

Patients in both groups were assessed for delayed postoperative complications to gain an insight into stomal granulation, stomal stenosis, tracheoesophageal fistula, tracheocutaneous fistula, tracheostomy tube blockage, stoma infection, and secondary hemorrhage. A significant variation (p≤0.05%) was observed in terms of stomal granulation, tracheostomy tube blockage, and stoma infection between the groups, with patients in the conventional group showing a higher prevalence, indicating that Bjork flap tracheotomy patients have fewer complications.

Tracheal or stomal stenosis is a dreadful complication of tracheotomy. The patients were followed up for tracheal stenosis at four months and it was observed that only two patients, one in each group, reported tracheal stenosis (p≥0.05). However, the incidence of tracheal stenosis has been reported to vary between 0 and 21% in various studies conducted earlier [19]. Similar findings have been reported earlier by Mukherjee et al., wherein none of the patients operated on by either procedure reported tracheal stenosis. However, the chances of tracheal or stomal stenosis are higher in conventional tracheotomy-treated patients. No cases have been documented in the flap tracheotomy group, owing to the early maturation and recovery of the tract. In comparison to the Bjork flap group, the conventional group has a higher risk of stoma injury as the inferior lip in the former procedure ensures ease of tube placement. Furthermore, the likelihood of peristomal granulation is lower in the flap-treated group as these granulations are less likely to block the flap-based mature stoma. Notably, the probability of stomal stenosis is aggravated and accentuated by immature healing of stoma through fibrosis, traumatizing peristomal tissue, and frequent or repetitive injury to granulation owing to tube placement [15]. Therefore, flap tracheotomy is much safer and aids in fast recovery. The findings are based on prior research suggesting that an inverted U-shaped flap preserves the tracheal cartilage and maintains the lumen following tracheotomy. The blood flow to the trachea is from an inferior direction, which ensures the survival of tracheal cartilage even after prolonged cannulation [20].

Decannulation was performed on 25 patients in our study: 15 in the conventional group and 10 in the Bjork flap group. Post-decannulation, the patients were followed up for six months. None of the patients developed any complications like tracheocutaneous fistula. Likewise, Malata et al. conducted a six-year retrospective study and reported that the inferiorly hinged (Bjork) flap tracheotomy technique can be safely used in adult patients undergoing head and neck cancer surgery to provide a secure airway. Furthermore, the study reported that no patient developed tracheal fistula, tracheal stenosis, or cosmetically unacceptable scarring, and there was no tracheotomy-related mortality. Thus, it is clear that the rates of occurrence of various complications were higher in patients treated with the conventional technique. These complications could be addressed to a larger extent by flap tracheotomy [10].

## Conclusions

Based on our findings, the rate of complications was significantly lower in Bjork flap tracheotomy as compared to conventional tracheotomy-treated patients. Hence, we recommend that surgeons choose the Bjork flap technique over the conventional procedure for elective tracheotomy in adults.
